# Supplementary material for: Serial dependence predicts generalization in perceptual learning
Source: eLife. 2026 Jul 20;15:RP109830. doi: 10.7554/eLife.109830 (PMC13384495; doi:10.7554/eLife.109830)
Supplement: MDAR checklist [file elife-109830-mdarchecklist1.docx]

**Materials Design Analysis Reporting (MDAR) Checklist for Authors**

The MDAR framework establishes a minimum set of requirements in transparent reporting mainly applicable to studies in the life sciences. eLife asks authors to provide detailed information within their article to facilitate the interpretation and replication of their work. For all that apply, please note where in the article the information is provided. Please note that eLife also collects information about data availability and ethics in the submission form.

| **Manuscript title** | Serial Dependence Predicts Generalization in Perceptual Learning |
| --- | --- |
| **Corresponding author** | Yoram S. Bonneh |
| **Manuscript number** | *109830* |

**Materials**

| **Requirement** | **Author response (where provided in article, or N/A)** |
| --- | --- |
| Newly created materials: a materials availability statement is included describing access and any restrictions. | N/A — secondary analysis of existing behavioural data; no new physical materials were created. |
| Antibodies: supplier, catalogue number, RRID. | N/A |
| DNA and RNA sequences: novel sequences included or deposited. | N/A |
| Cell materials: cell lines / primary cultures (species, strain, accession/RRID). | N/A |
| Experimental animals: laboratory/model organisms or field-captured (species, strain, sex, age, source). | N/A |
| Plants and microbes: species, strain, accession, source. | N/A |
| Human research participants: report age, sex, gender, ethnicity where collected. | Adult human observers (n = 50). See Materials and methods. *Demographics were not collected.* |

**Design**

| **Requirement** | **Author response (where provided in article, or N/A)** |
| --- | --- |
| Study protocol: if pre-registered, provide DOI; for clinical trials, registration number/DOI. | Not pre-registered. N/A. |
| Laboratory protocol: provide DOI/citation if step-by-step protocols available. | N/A |
| Experimental design – Sample-size determination: state whether/how done. | See Materials and methods. The study is a reanalysis of previously collected data (Harris, Gliksberg & Sagi, 2012). |
| Experimental design – Randomisation: state whether/how done. | Stimulus/trial order randomised (see Materials and methods); group assignment in Appendix 1—table 1. |
| Experimental design – Blinding: state whether/how done. | *Not applicable — secondary analysis of previously collected data (Harris, Gliksberg & Sagi, 2012); blinding pertained to the original data collection. Analyses in the present study were condition-aware by design.* |
| Experimental design – Inclusion/exclusion criteria: state whether/how done. | Trial-filtering criteria (e.g., SOA thresholds) described in Materials and methods; robustness without filtering shown in Appendix 1—figure 3. |
| Sample definition & in-lab replication: number of replications; technical vs biological replicates. | Replicates are individual human observers (n = 50; biological replicates). See Materials and methods and Appendix 1—table 1. |
| Ethics – human participants: authority granting approval and reference number. | Original data collection (Harris, Gliksberg & Sagi, 2012) received ethics approval; conducted per the Declaration of Helsinki with written informed consent. |
| Ethics – experimental animals: approval authority and reference number. | N/A |
| Ethics – specimen/field samples: permits/authority. | N/A |
| Dual Use Research of Concern (DURC): authority and reference number if applicable. | N/A |

**Analysis**

| **Requirement** | **Author response (where provided in article, or N/A)** |
| --- | --- |
| Attrition: whether exclusion criteria pre-established; data points omitted (attrition vs intentional) with justification. | Trial-level filtering criteria pre-established and described in Materials and methods; all data available on Dryad. |
| Statistics: describe statistical tests used and justify choice. | Described in Materials and methods (mixed-design ANOVA, post-hoc Tukey, orthogonal regression with bootstrap CIs, Pearson correlation). |
| Data availability: statement giving access details for newly created and reused datasets, with accession/DOI and licence. | Data availability statement: raw data openly available in Dryad, DOI 10.5061/dryad.69p8cz9jw. Reused behavioural data originate from Harris, Gliksberg & Sagi (2012) and are included in the deposit. |
| Code availability: statement giving access for code/software/algorithms essential to replicate findings, with accession/DOI and licence. | MATLAB analysis code for reproducing all figures is openly available in the same Dryad record (DOI 10.5061/dryad.69p8cz9jw). See Data availability statement. |

**Reporting**

| **Requirement** | **Author response (where provided in article, or N/A)** |
| --- | --- |
| Adherence to community standards: state if relevant guidelines (ICMJE, MIBBI, ARRIVE, STRANGE) followed and whether a checklist (CONSORT, PRISMA, ARRIVE) is provided. | N/A — no discipline-specific reporting guideline applies to this behavioural psychophysics reanalysis. |
