# Supplementary material for: Serial dependence predicts generalization in perceptual learning
Source: eLife. 2026 Jul 20;15:RP109830. doi: 10.7554/eLife.109830 (PMC13384495; doi:10.7554/eLife.109830)
Supplement: Peer review history

# Editor report: eLife Assessment

**DOI:** 10.7554/eLife.109830.3.sa0

**Authors:** Krystel R Huxlin

**Affiliations:** University of Rochester United States

**Keywords:** Compelling, Fundamental

This **fundamental** study describes long-range serial dependence of performance on a visual texture discrimination training task that manipulated conditions to induce differing degrees of location transfer of learning. The authors re-analyzed previously-published, behavioral data, generating **compelling** evidence from converging approaches that the serial dependence effects persist over multiple days of training, and may share a common causal mechanism with training-induced location transfer. By informing our understanding of the importance of temporal integration to long-term perceptual learning and its propensity towards specificity or generalizability, these results should interest neuroscientists who seek to uncover underlying neural mechanisms for these processes.

# Referee report: Reviewer #1 (Public review)

**DOI:** 10.7554/eLife.109830.3.sa1

This paper presents a reanalysis of a large existing dataset to examine whether serial dependence effects-systematic influences of recent stimulus history on current perceptual judgments-are associated with generalization in perceptual learning. The central hypothesis is that extended, longer-range history effects (beyond the most recent trials) are beneficial for transfer across locations. The authors reanalyze data from a texture discrimination task in which observers discriminated peripheral target orientation against a line background, with performance quantified by stimulus-onset asynchrony thresholds. Three training conditions were compared: a fixed single-location condition, a two-location alternating condition, and a dummy-trial condition with frequent target-absent trials. Transfer was assessed after training at new locations. Serial dependence was quantified using history-sequence analyses and linear mixed-effects models estimating bias weights across stimulus lags, with summary measures distinguishing recent (1-3 trials back) and more distant (4-6 trials back) dependencies.

The authors report extended serial dependence effects, persisting up to 6-10 trials back, with substantial cumulative bias that remains stable across multiple days of training and is not correlated with overall performance thresholds. Recent history effects are stronger for faster responses, suggesting a contribution from decision- or response-related processes, whereas more distant effects decline within sessions, potentially reflecting adaptation dynamics. Critically, longer-range serial dependence is significantly stronger in training conditions that promote generalization than in the single-location condition. Individual differences in the strength and decay profile of distant history effects predict the magnitude of transfer across locations, whereas recent history effects do not. History effects are also correlated across trained locations, suggesting stable individual differences.

The authors interpret longer-range serial dependence as reflecting integrative processes that extract task-relevant structure over time, thereby supporting generalization, while shorter-range effects are attributed to more transient mechanisms such as priming or decision-level bias. The discussion connects these findings to Bayesian accounts of perceptual stability and to concepts of overfitting in machine learning.

The study offers a novel and thoughtful link between short-term serial dependence and long-term generalization in perceptual learning, helping bridge two literatures that are often treated separately. The large dataset enables robust estimation of individual differences, and the use of mixed-effects modeling appropriately accounts for variability across observers. The empirical distinction between recent and more distant history effects is well-supported and adds important nuance to interpretations of serial dependence. Converging evidence from both group-level comparisons and individual-level correlations strengthens the central conclusions.

Comments on revisions:

The authors have effectively addressed my concerns. The new robustness analyses (Supp. Fig. S3), supplementary toy model, clearer DDM-based mechanistic distinctions, and expanded discussion of limitations and generality fully resolve my original points.

# Referee report: Reviewer #3 (Public review)

**DOI:** 10.7554/eLife.109830.3.sa2

Summary:

This reanalysis of a classic study of visual perceptual learning in a texture discrimination task convincingly demonstrates the presence of sequential dependence effects, commonly seen in response time analyses in 2-alternative tasks, on response accuracy in the texture task in visual periphery and in a simultaneous central letter report at fixation. Overall, this paper provides a new and interesting analysis of the effects of sequential dependencies from trial to trial on performance, learning, and generalizability in perceptual learning.

Strengths:

This new analysis of sequential dependency effects (SDEs) extends commonly observed sequential effects in two-choice reaction times to accuracy and relates them to response accuracy during visual learning in a frequently used perceptual learning task. The paper makes a convincing case that different conditions known to impact generalization of learning to a second visual location also expresses quantitatively distinct n-back SDEs.

Weaknesses:

Additional analyses now back up the analysis of effects of SDEs using trials selected to enhance the size of the effects, specifically when the current trial is low visibility and the prior trial is of high visibility. The authors now provide a practical analytic reason for this choice.

Comments on revisions:

The revision has successfully addressed comments in the original reviews.

# Author comment: Author response

**DOI:** 10.7554/eLife.109830.3.sa3

**Authors:** Noga Pinchuk Yacobi, Dov Sagi, Yoram Bonneh

**Affiliations:** Bar-Ilan University Ramat Gan Israel; Weizmann Institute of Sceince Rehovot Israel; Bar-Ilan University Ramat Gan Israel

The following is the authors’ response to the original reviews.

**Public Reviews:**

**Reviewer #1 (Public review):**

This paper presents a reanalysis of a large existing dataset to examine whether serial dependence effects-systematic influences of recent stimulus history on current perceptual judgments-are associated with generalization in perceptual learning. The central hypothesis is that extended, longer-range history effects (beyond the most recent trials) are beneficial for transfer across locations. The authors re analyze data from a texture discrimination task in which observers discriminated peripheral target orientation against a line background, with performance quantified by stimulus-onset asynchrony thresholds. Three training conditions were compared: a fixed single location condition, a two-location alternating condition, and a dummy-trial condition with frequent target-absent trials. Transfer was assessed after training at new locations. Serial dependence was quantified using history-sequence analyses and linear mixed effects models estimating bias weights across stimulus lags, with summary measures distinguishing recent (1-3 trials back) and more distant (4-6 trials back) dependencies.

The authors report extended serial dependence effects, persisting up to 6-10 trials back, with substantial cumulative bias that remains stable across multiple days of training and is not correlated with overall performance thresholds. Recent history effects are stronger for faster responses, suggesting a contribution from decision- or responserelated processes, whereas more distant effects decline within sessions, potentially reflecting adaptation dynamics. Critically, longer-range serial dependence is significantly stronger in training conditions that promote generalization than in the single-location condition. Individual differences in the strength and decay profile of distant history effects predict the magnitude of transfer across locations, whereas recent history effects do not. History effects are also correlated across trained locations, suggesting stable individual differences.

The authors interpret longer-range serial dependence as reflecting integrative processes that extract task-relevant structure over time, thereby supporting generalization, while shorter-range effects are attributed to more transient mechanisms such as priming or decision-level bias. The discussion connects these findings to Bayesian accounts of perceptual stability and to concepts of overfitting in machine learning.

The study offers a novel and thoughtful link between short-term serial dependence and long-term generalization in perceptual learning, helping bridge two literatures that are often treated separately. The large dataset enables robust estimation of individual differences, and the use of mixed-effects modeling appropriately accounts for variability across observers. The empirical distinction between recent and more distant history effects is well-supported and adds important nuance to interpretations of serial dependence. Converging evidence from both group-level comparisons and individuallevel correlations strengthens the central conclusions.

Several limitations should be addressed. First, the study relies entirely on previously collected data, without experimental manipulations designed to selectively isolate serial dependence mechanisms. Filtering choices, while theoretically motivated, may amplify history effects in ways that are difficult to quantify. Second, sequential dependencies can arise from multiple sources, including gradual updating of internal weight structures, adaptation processes, and history-dependent biases in decisionmaking. The current analyses do not clearly separate these contributions, limiting mechanistic attribution of long-range effects. Third, the conclusions are based on a single perceptual task, leaving open questions about generality across paradigms. Finally, while the discussion references computational ideas, no explicit modeling is provided to test whether plausible learning rules can jointly account for the observed history profiles and transfer effects.

We now address these issues in the manuscript (see below for detailed responses) and provide a toy model (supplementary material) where the observed effects are explained by simple learning mechanisms.

The findings align with theoretical frameworks that conceptualize perceptual learning as gradual reweighting of stable sensory representations at the decision stage (e.g., Petrov et al., 2005). Trial-by-trial updates in these models naturally give rise to sequential dependencies and sensitivity to training statistics. The observation that longer-range history effects predict generalization is consistent with broader temporal integration supporting more flexible learning, while narrower integration may lead to specificity. The results also indicate that multiple mechanisms - including decisionlevel biases and adaptation - may coexist with reweighting processes, highlighting the value of hybrid accounts.

In summary, this is a careful and data-rich reanalysis that highlights a potentially important role for serial dependence in enabling generalization during perceptual learning. While the underlying mechanisms remain underspecified, the evidence supporting the reported associations is strong, and the work provides a valuable empirical foundation for further experimental and modeling efforts.

**Reviewer #2 (Public review):**

This manuscript investigates how people's perceptual reports are influenced by events and trials in the past, and how this long-range dependence relates to broader learning across locations in a visual learning task. The authors present clear and internally consistent analyses showing that extended temporal integration is associated with greater generalization of learning. The study is thought-provoking and may contribute meaningfully to understanding how short-term influences and long-term improvement interact, although several interpretational points would benefit from clarification.

Strengths:

(1) The manuscript identifies unusually long-range perceptual biases extending up to ten trials back, which is a striking and potentially important finding.

(2) The association between strong long-range dependence and greater learning generalization is clearly documented and supported by consistent analyses.

(3) The dataset is large and rich, and the authors apply repeated and well-controlled analyses that give confidence in the stability of the effects.

(4) The writing is generally clear, and the manuscript raises interesting conceptual links between temporal integration and generalization of learning.

Weaknesses / Points Requiring Clarification:

(1) The manuscript repeatedly equates generalization with increased efficiency, but this relationship is not universally true. In some populations or tasks, excessive generalization can reduce task-specific efficiency. The authors should discuss this context-dependence to clarify when generalization is beneficial versus detrimental.

We agree with the reviewer that generalization does not strictly imply increased efficiency; in some contexts, over-generalization can indeed be detrimental. We now explicitly note in the Introduction that serial dependence can impair performance when stimuli vary randomly across trials. We have reviewed the manuscript to ensure we do not explicitly equate generalization with efficiency. Our argument is specifically that long-range SDEs support the transfer of learning (generalization).

(2) Serial dependence is also present, though smaller, in the central fixation task. It remains unclear whether this bias could contribute to the serial dependence observed in the main task. The authors should clarify whether the two biases are independent or whether the central-task bias might partially influence orientation judgments in the main task.

These two tasks are independent, one requires T/L discrimination the other V/H discrimination. See our detailed response below.

(3) Several figure captions and labels contain minor inconsistencies in formatting and terminology. Careful proofreading would improve clarity.

We thank the reviewer for pointing this out and have proofread the captions to improve formatting and terminology consistency throughout.

**Reviewer #3 (Public review):**

This reanalysis of a classic study of visual perceptual learning in a texture discrimination task convincingly demonstrates the presence of sequential dependence effects, commonly seen in response time analyses in 2-alternative tasks, on response accuracy in the texture task in the visual periphery and in a simultaneous central letter report at fixation. Overall, this paper provides a new and interesting analysis of the effects of sequential dependencies from trial to trial on performance, learning, and generalizability in perceptual learning.

Strengths:

This new analysis of sequential dependency effects (SDEs) extends commonly observed sequential effects in two-choice reaction times to accuracy and relates them to response accuracy during visual learning in a frequently used perceptual learning task. The paper makes a convincing case that different conditions known to impact generalization of learning to a second visual location also express quantitatively distinct n-back SDEs.

Weaknesses:

Most of the new analyses emphasize the effects of SDEs, including trials designed to enhance the size of the effects, specifically when the current trial is low visibility, and the prior trial is of high visibility. Unless there is an argument that learning and subsequent generalization primarily occur in low-visibility trials, the presentation should also include displays and an emphasized discussion of analysis for all trials, unfiltered.

We analyze effects on close to threshold (small-medium SOA) current targets preceded by above threshold (high SOA) reference targets. This is motivated by both technical issues and theoretical assumptions. In psychophysics, when using percent correct as a measure of performance, bias cannot be reliably estimated at or near ceiling performance, as correct responses leave little room for bias to manifest. Regarding the ‘easy’ targets used as a reference, having them at low SOA introduces uncertainty as for the reference orientation against which bias is measured, with their perceptual effect being ambiguous. Theoretically, we note that in perceptual learning with threshold targets, the introduction of clear targets in the absence of feedback enables learning (see Discussion, where we added: 'Most interestingly, in our experiments without feedback on the texture task, the experimental conditions yielding the strongest bias were also found to enhance learning in the absence of feedback (Liu et al., 2012)')

We have addressed this concern also by conducting additional robustness analyses with unfiltered prior-trial history. We analyzed data without the prior-visibility filter; results are presented in a new Supplementary Figure S3 and confirm our main findings (see addition to Methods: "Finally, to verify that our findings are not artifacts of these filtering choices, we also conducted control analyses including all prior-trial history regardless of visibility; these results are presented in Supplementary Figure S3 and confirm the robustness of our main findings.").

**Recommendations for the authors:**

**Reviewer #1 (Recommendations for the authors):**

(1) How manipulations of stimulus statistics, uncertainty, or feedback could selectively engage different forms of serial dependence

We expect serial dependence to be modulated by all these parameters. In classical SDT, stimulus statistics are known to affect response bias, as are temporal correlations in stimulation sequences. We note in the manuscript that we employed random sequences (50% chance for V and 50% for H targets), eliminating expectation-based biases toward either orientation. Stimulus uncertainty is known to increase serial dependence, as we also found here. Feedback is also expected to have an effect, the literature is somewhat ambiguous about this, but this may also depend on experimental design. We note that the main task studied here (TDT) had no feedback while the central T/L task did have feedback, both showing serial dependencies. In the manuscript we point to reviews of SDE where much of this is discussed.

(2) How explicit computational models could help distinguish decision bias from structural learning

We use the drift diffusion model (DDM) to distinguish decision bias (starting point in DDM) from structural learning (changes in drift rate). DDM predicts that decision bias is short lived, mainly affects fast reaction times (RT) while biases due to drift rate asymmetry persists to long RTs. We present these results in Figure 3.

(3) Whether similar relationships are observed in other perceptual domains

We are not aware of any other study linking serial dependence and perceptual learning or reporting such a link. We expect the link between long-range serial dependence and learning generalization to extend beyond the TDT (see new paragraph in Discussion). We hope this framework will motivate similar analysis in other labs where comparable datasets exist.

(4) How sensitive are the results to the filtering choices used in the analysis?

We analyze effects on close to threshold (small-medium SOA) current targets preceded by above threshold (high SOA) reference targets. This is motivated by both technical issues and theoretical assumptions. In psychophysics, when using percent correct as a measure of performance, bias cannot be reliably estimated at or near ceiling performance, as correct responses leave little room for bias to manifest. Regarding the ‘easy’ targets used as a reference, having them at low SOA introduces uncertainty as for the reference orientation against which bias is measured, with their perceptual effect being ambiguous. Theoretically, we note that in perceptual learning with threshold targets, the introduction of clear targets in the absence of feedback enables learning (see Discussion, where we added: 'Most interestingly, in our experiments without feedback on the texture task, the experimental conditions yielding the strongest bias were also found to enhance learning in the absence of feedback (Liu et al., 2012)')

We have addressed this concern also by conducting additional robustness analyses with unfiltered prior-trial history. We analyzed data without the prior-visibility filter; results are presented in a new Supplementary Figure S3 and confirm our main findings (see addition to Methods: "Finally, to verify that our findings are not artifacts of these filtering choices, we also conducted control analyses including all prior-trial history regardless of visibility; these results are presented in Supplementary Figure S3 and confirm the robustness of our main findings.").

**Reviewer #2 (Recommendations for the authors):**

(1) Clarify mechanisms underlying long-range serial dependence. Please better distinguish possible sources of serial dependence (e.g., decision bias, adaptation, reweighting) and clarify which interpretations are supported or remain ambiguous given the current analyses

Our manuscript discusses the mechanisms underlying the dissociation between recent and distant SDEs in the Discussion section. Specifically, we report that:

Recent SDEs are RT-dependent (stronger with faster responses) consistent with decision-level criterion shifts (Dekel & Sagi, 2020)

Distant SDEs are RT-independent consistent with neural reweighting/template updating

We also discuss the role of sensory adaptation in truncating long-range integration, supported by within-session decline of SDEs, reduced distant SDEs in the 1loc condition, and the original findings by Harris et al. (2012).

We have added an explicit acknowledgment that our correlational approach cannot definitively establish causality (see addition to Discussion: "While these converging findings support distinct mechanisms for recent and distant SDEs, our correlational approach cannot definitively establish causality, and targeted experimental manipulations would further strengthen these interpretations.").

(2) Test robustness to analytic choices

We have conducted robustness analyses by removing the prior-trial visibility filter. The results are presented in a new Supplementary Figure S3 and confirm that our key findings remain qualitatively unchanged (see addition to Methods referencing Supplementary Figure S3).

(3) Strengthen the computational link

We have expanded the Discussion to reference relevant computational models and specify predictions for future modeling work. We now cite Petrov et al. (2005). We provide a toy model implementing trial-by-trial template update that show SDE that is correlated with learning transfer. Importantly, in this model, long range SDE is a consequence of learning dynamics (see new paragraph in Discussion, and model simulation in supplementary material).

(4) Discuss generality and experimental tests. Briefly address whether similar effects are expected across other tasks or sensory domains, and outline experimental manipulations that could causally test the role of serial dependence in generalization.

We have added discussion of generality across perceptual domains and outlined the prediction that future work could test the SDE-generalization link in other tasks where both phenomena have been documented (see new paragraph in Discussion).

Reviewer #2 (Public Review - Point 2): Central task SDE independence

The SDEs observed in the central letter task and peripheral TDT are likely independent, as they involve different stimulus features (letter identity vs. orientation), different response mappings, and show distinct performance patterns across conditions. The absence of condition differences in central-task SDEs (described in the Results section under "SDE differences between conditions" end of paragraph), despite robust differences in TDT SDEs, further suggests that the peripheral orientation biases are not contaminated by central-task response tendencies. Note that the central task was fixed across conditions, stayed at fixation when location was changed, and when dummy trials were presented.

**Reviewer #3 (Recommendations for the authors):**

(1) Reference to Falmagne, Cohen, & Dwivedi (1975)

We have added this reference to the Introduction, acknowledging the historical foundation of sequential effects in perceptual decisions

(2) The SDE data of Figure 1 are (per the figure legend) from the 1 loc data of Harris et al., "pooled over all testing days", and filtered for trials with low-visibility current targets (SOA < SOA-threshold+20ms). Specify whether this threshold criterion is on a per-subject basis. State in the legend that "all testing days" includes Days 1-8 (4 days with the first location and another 4 days testing generalization to a second location).

We have revised the Figure 1 legend to clarify:

"Days 1–8; 4 days at the first location and 4 days at the second location to assess generalization"

"calculated on a per-subject basis"

(3) The leadup emphasizes that the analysis in the figure emphasizes trials where the effect is expected to be as large as possible (cited as 40 +/- 3%), while visible current targets (at n) biases were 5+/-1%.

See below, after (4).

(4) Unless a theoretical position associates learning just with low visibility (if so, explain), consider including two other panels showing the sequential dependencies for all trials, and the linear model weights over the last 10 trials for all trials.

We acknowledge that the main analyses emphasize conditions that maximize SDE expression. To verify robustness, we conducted control analyses including all prior-trial history regardless of visibility; these results are presented in Supplementary Figure S3 and confirm our main findings.

There are both theoretical and technical justifications for the filtering applied:

It is well known that learning, in particular without feedback (as in our TDT), is facilitated by a mixture of threshold level stimuli and suprathreshold easy trials (e.g., Liu et al., 2012).

Technically, it is impossible to measure bias with highly discriminable stimuli where performance is perfect or close to it, thus such trials are expected to dilute the measured effect. On the other hand, when considering serial effects from low sensitivity trials, we face an uncertainty involved in defining the actual orientation relative to which the bias needs to be computed.

(5) Figure S1 seems to indicate that average thresholds over all days (location 1 and location 2) are unrelated to the sequential dependence across subjects and that the amount of learning in location 1 is unrelated to the sequential dependencies across subjects in all the varied conditions. Since Figure S1 includes all 50 subjects, it includes some conditions with dummy trials interspersed. Clarify in the description whether the dummy trials are ignored for the purposes of the SDE analyses.

We have clarified in the Methods how trials are handled in the analysis: "To preserve the precise temporal structure of the data, all trials were included in the sequential n-back count across all experimental conditions, thus dummy trials were counted as time bins but their contribution was ignored. In the Linear Mixed Effects (LME) analysis, we modeled these trial types using distinct regressors: each n-back lag included separate predictors for visible and invisible targets, further differentiated by trial type (dummy vs. target) and relative location (ipsilateral vs. contralateral) where applicable. The SDE values reported here reflect only the influence of relevant target-present history trials; the effects of other history types (e.g., dummy trials), while estimated to ensure the temporal integrity of the model, are not presented."

(6) The conclusion from this analysis seems to be that the overall average threshold and the amount of initial learning are both uncorrelated with the strength of sequential dependencies across subjects. This conclusion should be added to the description in the main paper.

This finding is now discussed in the Discussion section, referring to the main Results section [No significant correlation was found between biases and SOA thresholds across observers (r = -0.13, p = 0.37, average across days 1-8), nor between biases and improvements in performance at the first location (r = -0.09, p = 0.54, average across days 1-4), suggesting that the magnitude of serial dependence does not predict the overall amount of perceptual learning (Supplementary Figure S1)].

(7) Decay of SDE section clarifications

We have made the following clarifications:

RT definition: Added to Methods: "The reaction time (RT) used in the analysis was defined as RT(TDT) – RT (fixation task), where RT for each task was measured from stimulus onset."

N-back counting: Clarified in Methods (see response to point 5 above): all trials were included in the chronological sequence; the LME analysis assigned separate predictors at each lag for visible/invisible targets and for trial categories (dummy vs. target) and locations (ipsilateral vs. contralateral). The results reported do not include effects of dummy trial, except where response dependent SDE was reported (Fig 2a, SDE for response key).

2loc n-back effect: The longer-range effects in the 2loc condition likely reflect reduced adaptation allowing longer temporal integration, combined with the location-selective nature of SDEs.

RT and mechanism interpretation: The manuscript discusses that the critical observation is the qualitative difference in RT sensitivity between recent and distant SDEs, consistent with the drift-diffusion framework where criterion shifts are RTdependent while drift bias is RT-independent (Dekel & Sagi, 2020). We have added an acknowledgment of the correlational limitations of this interpretation.

Moving figures to supplement: We prefer to keep Figures 4 and 5 in the main text as they document important dynamics supporting our mechanistic interpretation.
